# The Relationship between Crude Oil Futures Market and Chinese/US Stock Index Futures Market Based on Breakpoint Test

**DOI:** 10.3390/e23091172

**Published:** 2021-09-06

**Authors:** Xunfa Lu, Kai Liu, Kin Keung Lai, Hairong Cui

**Affiliations:** 1 School of Management Science and Engineering, Nanjing University of Information Science and Technology, Nanjing 210044, China; 002395@nuist.edu.cn; 2 Center for Economics, Finance and Management Studies, Hunan University, Changsha 410006, China; liukaiedu@hnu.edu.cn; 3 School of Economics and Commerce, Guangdong University of Technology, Guangzhou 510006, China

**Keywords:** stock index futures, WTI crude oil futures, transmission relationship, breakpoint test, VAR–DCC–GARCH

## Abstract

Combined with the B-P (breakpoint) test and VAR–DCC–GARCH model, the relationship between WTI crude oil futures and S&P 500 index futures or CSI 300 index futures was investigated and compared. The results show that breakpoints exist in the relationship in the mean between WTI crude oil futures market and Chinese stock index futures market or US stock index futures market. The relationship in mean between WTI crude oil futures prices and S&P 500 stock index futures, or CSI 300 stock index futures is weakening. Meanwhile, there is a decreasing dynamic conditional correlation between the WTI crude oil futures market and Chinese stock index futures market or US stock index futures market after the breakpoint in the price series. The Chinese stock index futures are less affected by short-term fluctuations in crude oil futures returns than US stock index futures.

## 1. Introduction

It is well-known and accepted that crude oil is “the blood of industry” and an important commodity in international trade. Therefore, investigation and understanding of fluctuations in prices of crude oil plays a decisive role in smooth operations of the entire international trade. At the same time, because crude oil price and its derivatives are increasingly viewed as part of investment portfolios by financial market participants, it is particularly important to study their relationship with different financial markets. Baum et al. [1] studied characteristics of the volatility of WTI crude oil futures, natural gas futures and S&P 500 stock index futures based on high-frequency data, and found that introduction of leverage and jumps in return volatility models can improve accuracy of volatility prediction. Shaeri and Katircioğlu [2] used the multiple regime shifts model to study the long-term equilibrium relationship between crude oil prices and prices of stocks of oil-related enterprises and confirmed that crude oil prices have a significant positive impact on stocks of oil-related enterprises. Liu et al. [3] studied the relationship between implied volatility of the international crude oil market and the US stock market and found that this relationship changes significantly with changes in crude oil and stock market policies. Wu et al. [4] used the structural break test and VAR model to analyze correlations between carbon trading, crude oil and stock markets and found that there is significant bidirectional nonlinear Granger causality among the three markets and that they are causes and advancements of each other.

Crude oil futures were developed to help market participants manage risks and respond to fluctuations in crude oil prices. Futures products are popular among investors because of their special hedging function, enabling short selling and use of high leverage. Luo et al. [5] used the infinite hidden Markov model to predict fluctuations of international crude oil futures under the condition of introduction of exogenous variables. Wang and Hou [6] and Li et al. [7] provided evidence that the Chinese crude oil futures play a good role in facilitating hedging risks. At the same time, as a barometer of the overall economic performance of the market, stock index futures can help investors avoid investment risks, reduce stock market volatility and diversify investment strategies. A large number of policy makers, practitioners and scholars have paid attention to the research on relationships between stock indices and futures markets [8,9,10]. In particular, the return and volatility characteristics of S&P 500 stock index futures, as one of the most traded stock index futures in the world, have always been the focus of attention of the majority of market participants. For example, Martens [11] measured the volatility of S&P 500 stock index futures based on high-frequency data. Hamid and Iqbal [12] used the neural network to improve the accuracy of prediction of the volatility of S&P 500 stock index futures. By studying the trading behavior of S&P 500 stock index futures, Smales [13] found that after the 2008 financial crisis, investors abandoned the previously widely adopted positive feedback trading method and switched to the investment strategy with market volatility and investor sentiment as the main reference standard. China, which has the world’s second largest economy, has been developing stock index futures for a relatively short time, but it is still widely followed by financial market participants. Xie and Huang [14] found that the launch of CSI 300 index futures did not reduce volatility of the spot market. They employed a set of GARCH models to investigate the impact of index futures trading on volatility of the spot market in China. Miao et al. [15] showed that there were significant return and volatility shocks transmitted from the Chinese stock market to the CSI 300 index futures market. Chen and Gong [16] made a comparative study of the relationship between the futures and spot markets of the CSI 300 stock index before and after 2015, and concluded that futures were not the main influencing factors of the stock market crash in 2015.

The relationship between crude oil and stock markets has been widely studied [17,18,19], but most of these studies have focused on spot markets; few studies have analyzed the correlation between futures prices in the two markets. This study selected stock index futures in the United States and in China as the research objects. One is the most powerful economy in the developed world, and the other is the most powerful economy among developing countries. Their market environments have big differences. The study of the futures market and crude oil futures market not only has vital significance for the two countries but also for the economy of the entire world.

There are mature research paradigms on the relationship between different markets, which can be divided into two aspects: mean transmission and volatility spillover. This paper follows this research paradigm by using VAR model to study mean transmission, supplemented by Granger causality test [20,21], and then using DCC–GARCH model to depict the fluctuation spillovers and the dynamic correlation coefficients [22]. In order to further describe the changes in the structure of the financial market, following Yu et al. [23] and Aloui et al. [24], this paper uses the B-P (breakpoint) test, VAR model and DCC–GARCH model to compare and investigate the dynamic transmission relationship between crude oil futures and stock index futures in China and the United States. Then it compares and analyzes the different results obtained in the overall sample and in the phased samples. Compared with the traditional research methods, the breakpoint test was used for multiple regressions of the unchanged mean model on the whole interval according to the structural breakpoint, so as to obtain the different mean characteristics in different periods. This can deeply depict the influence of the transformation of financial market structure on the two different markets. In addition, inspired by Reference [25] and allowing for the potential nonlinear relationship between crude oil futures and stock index futures in China and the United States, this paper also uses the maximal information coefficient (MIC) to identify the various relationships between crude oil futures and stock index futures as an alternative to the traditional Pearson linear coefficient. Note that the MIC is constructed by way of mutual information of the two random variables, which is the relative entropy between their joint distribution and their product distribution [26]. Finally, the residual information of VAR model is input into the DCC–GARCH model, and then the structural change of the coefficients of the dynamic correlation coefficient between crude oil futures and stock index futures is also examined based on the B-P (breakpoint) test.

The remainder of this paper is organized as follows. Section 2 is a description of the data and summary statistics, and Section 3 introduces the methodologies. The results are presented in Section 4, and Section 5 concludes the paper.

## 2. Data and Descriptive Statistics

The New York Exchange has the largest trading volume in the world, and it is extensively influenced by the West Texas Intermediate (WTI) crude oil futures. Therefore, WTI is selected as the representative of traded products in the crude oil futures market. The WTI crude oil futures contract has good liquidity and price transparency, so a large number of financial market participants regard it as an important part of portfolios. At the same time, it is also the main sample data selected by the academics to study oil-related issues and phenomena. It is well-known that the United States is the most representative developed country in the world and China is the most representative developing country. Therefore, stock index futures are chosen from their respective financial markets. From the US, S&P 500 index futures are selected as the representative of the American futures market. The S&P 500 gives a good picture of the US economy. Therefore, the futures contract based on it can also effectively reflect capital flows in and out of the American market. CSI 300 index futures are selected as the representative of China futures market. The CSI 300 is a fundamental indicator of the Chinese stock market. It can not only provide guidance on investment portfolios, but also provide reference standards for fund managers’ performance appraisal. Therefore, index futures underlying the CSI 300 index can truly reflect the expectations of market participants on China’s stock market. Data are obtained from China Stock Market & Accounting Research Database (http://www.gtarsc.com/, accessed on 6 September 2021) and Investing (https://cn.investing.com/, accessed on 6 September 2021). Since there are multiple delivery dates for the same type of futures, in order to make the data as consistent as possible, futures prices selected in this paper take the futures quotation closest to the delivery time that is finalized as the futures price of the same day.

**Figure 1:**
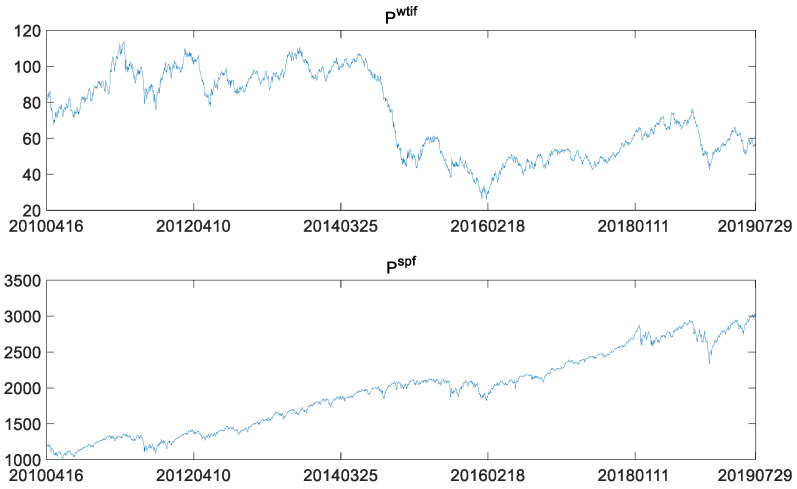
WTI crude oil futures prices and the S&P 500 futures prices.

Data of a total of 2408 observations were obtained by matching the WTI crude oil futures price date with the S&P 500 futures price data by date from 16 April 2010 to 29 July 2019. $P^{wtif}$ denotes the WTI crude oil futures prices, and $P^{spf}$ for S&P 500 stock index futures price. Fluctuations in these two price series are depicted in Figure 1.

Figure 1 clearly shows that WTI crude oil futures plummeted between 2014 and 2016, which is different from the almost steady rise of S&P 500 futures. Therefore, it is necessary to conduct an in-depth study on whether there were structural changes in WTI crude oil futures. Meanwhile, it is necessary to further analyze whether the transmission relationship between the WTI crude oil futures market and the S&P 500 stock index futures experienced structural changes when WTI crude oil futures experienced the structural changes.

Generally speaking, since prices of financial assets are not the stable data required by the time series model, the prices are converted into log-returns: $R_{t}^{wtif}=\log(P_{t}^{wtif})-\log(P_{t-1}^{wtif})$, $R_{t}^{spf}=\log(P_{t}^{spf})-\log(P_{t-1}^{spf})$, where $R^{wtif}$ and $R^{spf}$ indicate the log-returns of WTI crude oil futures and S&P 500 index futures. The plot of the two log-returns is as follows.

**Figure 2:**
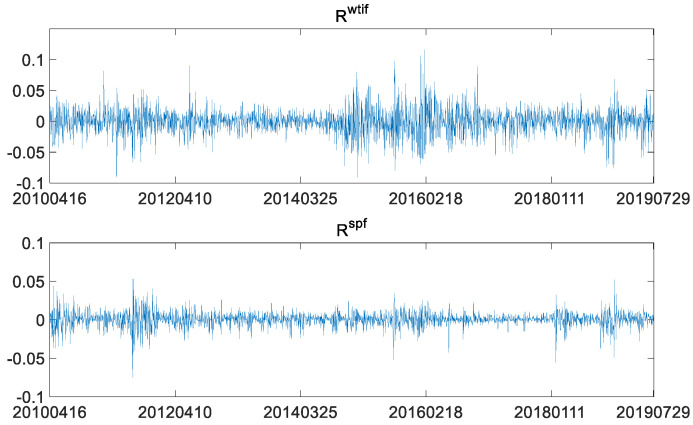
WTI crude oil futures log-returns and the S&P 500 index futures log-returns.

As shown in Figure 2, there is an obvious volatility clustering between log-returns of WTI crude oil futures and log-returns of S&P 500 stock index futures. That is, large fluctuations are often followed by large fluctuations, while small fluctuations are often followed by small fluctuations. Therefore, it is necessary to use a heteroscedasticity model, such as a GARCH-type model, to depict the characteristics of fluctuations in the two markets. Descriptive statistics of log-returns of the two futures are as follows.

It can be seen from Table 1 that ADF and PP tests of log-returns in both markets reject the null hypothesis of a unit root at 1% significance level, indicating that the log-returns are stationary, integrated in the same order of 0 lag, viz I(0), and suitable for long memory tests. The kurtosis values are larger than 3, showing that the log-returns’ series are leptokurtic. The JB statistics reject the null hypothesis of normality. It is worth noting that the mean of log-returns of WTI crude oil futures is less than 0; that is, the overall crude oil futures prices have declined since 2010. The downward trend in crude oil prices is also illustrated in Figure 1. Meanwhile, the S&P 500 stock index futures show the opposite trend. Log-returns of WTI crude oil futures are skewed to the right, while log-returns of S&P 500 index futures are skewed to the left.

A total of 2246 observations were obtained by matching the WTI crude oil futures price data with the futures price data of CSI 300, for the time window from 16 April 2010 to 23 August 2019. As mentioned above, $P^{wtif}$ indicates WTI crude oil futures price, and $P^{csif}$ indicates the price of CSI 300 futures.

**Figure 3:**
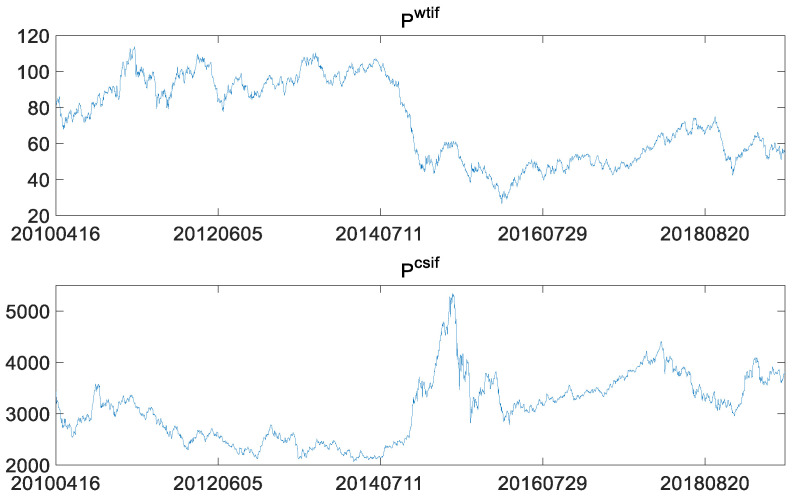
WTI crude oil futures prices and the CSI 300 futures prices.

As shown in Figure 3, fluctuations in CSI 300 stock index futures are different from WTI crude oil futures and in S&P 500 stock index futures, showing an obvious sharp rise and then fall. This is closely related to the weak and less efficient market in China, as it is one of the developing countries. The irrational behavior of market participants, the asymmetry of market information and the dependence of financial market on policies together contribute to the trends in CSI 300 stock index futures. Let the log-returns of CSI 300 futures be $R_{t}^{csif}=\log(P_{t}^{csif})-\log(P_{t-1}^{csif})$. The plot of the two log-returns is as follows.

**Figure 4:**
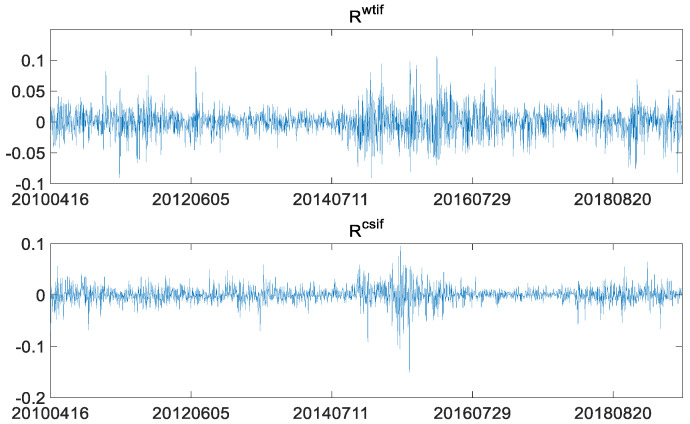
WTI crude oil futures log-returns and the CSI 300 index futures log-returns.

Similar to Figure 2, Figure 4 also shows clustering of volatility of log-returns in the two markets. For identifying the heteroscedasticity, the GARCH family models need to be considered. Descriptive statistics of the log-returns of the two futures are as follows.

Table 2 shows that the ADF and PP tests of log-returns in both markets also reject the null hypothesis of a unit root at 1% significance level, indicating that log-returns of CSI 300 stock index futures are as stationary and integrated in the same order of 0 lag, viz I(0), as those of WTI crude oil futures, and are suitable for long memory tests. Log-returns of CSI 300 stock index futures also have the characteristics of peakedness and fat tail. However, in terms of skewness, log-returns of CSI 300 stock index futures are different from those of WTI crude oil futures and similar to those of S&P 500 stock index futures, which too are left-skewed. The JB statistics also reject the null hypothesis of normality.

## 3. Research Methods

The Granger causality test was first used to identify the potential causal relationship between WTI crude oil futures and the two stock indices futures, and then the VAR model was applied to study the transmission relationship in mean between crude oil futures and the two stock indices futures. Because the Granger causality test and the VAR model can identify only the linear relationships between different variables, and cannot detect any nonlinear relationship, the MIC was further used to uncover the nonlinear relationship seen in the real world. Fourthly, the DCC–GARCH model was used to describe the correlation in volatility. Finally, in order to allow for the structural change in the financial markets, the B-P (breakpoint) test model was used to divide the pair-wise price series and dynamic conditional correlations obtained by DCC–GARCH model into groups. Each phased group was investigated by using the Granger causality test, VAR model and the MIC to obtain the transmission relationship in mean between markets. The dynamic conditional correlations between different groups (if any) are here compared and analyzed. This is beneficial to reveal the effect of financial market structural changes on the transmission relationship between WTI crude oil futures and stock index futures.

### 3.1. Granger Causality Test

If there is a causal relationship between two markets, then the previous information of the market as a cause must have an impact on the current value of the market. The granger causality test model is introduced by taking log-returns of WTI crude oil futures $R^{wtif}$ and S&P 500 stock index futures $R^{spf}$ as an example. The form of the model is as follows:

(1) $$R_{t}^{wtif}=\alpha_{0}+\alpha_{1}R_{t-1}^{wtif}+\cdots +\alpha_{p}R_{t-p}^{wtif}+\varepsilon_{t}^{wtif}$$

(2) $$R_{t}^{wtif}=\alpha_{0}+\alpha_{1}R_{t-1}^{wtif}+\cdots +\alpha_{p}R_{t-p}^{wtif}+\beta_{1}R_{t-1}^{spf}+\cdots +\beta_{p}R_{t-p}^{spf}+\varepsilon_{t}^{wtif}$$

The F statistic is constructed to examine whether $R^{spf}$ has a granger causality on $R^{wtif}$. If the F statistic is significant, it means there is a significant granger causal relationship from $R^{spf}$ to $R^{wtif}$. Moreover, α and β are the corresponding coefficients, and p represents the lag orders [21].

### 3.2. Vector Autoregressive Model

VAR model can accurately capture the transmission relationship in the mean between different markets [20]. Taking the log-returns of WTI crude oil futures and S&P 500 stock index futures as an example to introduce the specific form of VAR model, we get the following:

(3) $$R_{t}^{wtif}=c^{wtif}+\sum_{i=1}^{p}\phi_{t-i}^{wtif}R_{t-i}^{wtif}+\sum_{j=1}^{p}\theta_{t-j}^{spf}R_{t-j}^{spf}+\varepsilon_{t}^{wtif}$$

(4) $$R_{t}^{spf}=c^{spf}+\sum_{i=1}^{p}\phi_{t-i}^{wtif}R_{t-i}^{wtf}+\sum_{j=1}^{p}\theta_{t-j}^{spf}R_{t-j}^{spf}+\varepsilon_{t}^{spf}$$

where c is a constant, ε represents the random disturbances corresponding to the two markets and ϕ and θ represent the parameter vectors. If the value of ϕ is not significantly different from 0 in Equation (3), it means that $R^{wtif}$ is not statistically affected by its previous information. If significant, it is affected by its previous information. If the value of ϕ is not significantly different from 0 in Equation (4), it indicates that the previous information of $R^{wtif}$ has no significant influence on $R^{spf}$.

### 3.3. Maximal Information Coefficient

According to [25,27], the two random variables, X and Y, have a joint probability mass function, p(x, y), and marginal probability mass functions, p(x) and p(y), respectively. The mutual information, I(X, Y), is the relative entropy between the joint distribution and the product distribution, p(x)p(y):

(5) $$\begin{array}{ll} I\left(X,\;Y\right) & =H\left(X\right)+H\left(Y\right)-H\left(X,\;Y\right)\\ & =\sum_{i=1}^{n_{x}}p\left(x_{i}\right)\log_{2}\frac{1}{p\left(x_{i}\right)}+\sum_{j=1}^{n_{y}}p\left(y_{j}\right)\log_{2}\frac{1}{p\left(y_{j}\right)}-\sum_{i=1}^{n_{x}}\sum_{j=1}^{n_{y}}p\left(x_{i},\;y_{j}\right)\log_{2}\frac{1}{p\left(x_{i},\;y_{j}\right)} \end{array}$$

where $n_{x}$ and $n_{y}$ are the numbers of bins of the partition of the *x*- and *y*-axis. H(X) and H(Y) are the entropies of X and Y, respectively. H(X, Y) are the joint entropy of a pair of random variables (X, Y).

The MIC between X and Y is described by the following formula:

(6) $$MIC\left(X,\;Y\right)=\max\left\{I\left(X,\;Y\right)/\log_{2}\min\left\{n_{x},\;n_{y}\right\}\right\}$$

An MIC of zero indicates that there is no dependence between the considered random variables, while an MIC of one implies a stronger relationship. In this study, we assign X to $R^{wtif}$, and Y to $R^{spf}$ or $R^{csif}$.

### 3.4. DCC–GARCH Model

Based on the CCC (Constant Conditional Correlation) model proposed by Bollerslev [28], Engle [22] proposed the DCC (Dynamic Conditional Correlation) model. The DCC model drops the assumption of CCC model that the coefficient of correlation between variances remains unchanged, so that the correlation coefficient changes with time. The basic form of the DCC–GARCH model is as follows:

(7) $$\varepsilon_{t}∼N(0,H_{t}R_{t}H_{t})$$

(8) $$H_{t}^{2}=diag\{\omega_{i}\}+diag\{\kappa_{i}\}∙\varepsilon_{t-1}\varepsilon_{t-1}^{\prime }+diag\{\lambda_{i}\}∙H_{t-1}^{2}$$

(9) $$\eta_{t}=H_{t}^{-1}\varepsilon_{t}$$

(10) $$Q_{t}=S∙(\mu^{\prime }-\alpha -\beta )+\alpha ∙\eta_{t-1}\eta_{t-1}^{\prime }+\beta ∙Q_{t-1},\alpha +\beta <1$$

(11) $$R_{t}=diag{\left\{Q_{t}\right\}}^{-1}Q_{t}diag{\left\{Q_{t}\right\}}^{-1}$$

In Equation (7), $\varepsilon_{t}$ is the residual obtained from the VAR model and follows the normal distribution with 0 mean and heteroscedasticity; Equation (8) represents the GARCH(1,1) model, and $\eta_{t}$ is the standardized residual. Equation (10) is the unconditional correlation matrix of standardized residuals, where α and β are the DCC model’s coefficients. Moreover, α represents the short-term impact of the variances, and β represents the effect of previous variance on the current variance, viz the long-term impact. Equation (11) is the dynamic correlation coefficient matrix. The coefficient of correlation between variables i and j in time (t) is as follows:

(12) $$\rho_{i,j,t}=\frac{q_{i,j,t}}{\sqrt{q_{i,i,t}q_{j,j,t}}}$$

### 3.5. B-P (Breakpoint) Test

The B-P (breakpoint) test proposed by Bai and Perron [29] was used to identify the structural breakpoints of WTI crude oil futures and stock index futures. The B-P (breakpoint) test can select the breakpoints through the endogenous data, without human discretion, which avoids the problem of subjectivity.

The B-P (breakpoint) test assumes that there are m structural breakpoints in the data, that is, the data can be divided into m+1 intervals.

(13) $$\begin{array}{l} y_{t}=x_{t}^{\prime }\beta +z_{t}^{\prime }\delta_{1}+\mu_{t},t=1,\cdots ,T_{1}\\ y_{t}=x_{t}^{\prime }\beta +z_{t}^{\prime }\delta_{1}+\mu_{t},t=1,\cdots ,T_{1}\\ \vdots \\ y_{t}=x_{t}^{\prime }\beta +z_{t}^{\prime }\delta_{m+1}+\mu_{t},t=T_{m}+1,\cdots ,T \end{array}$$

where $y_{t}$ represents the time series variables; $x_{t}$ and $z_{t}$ are the explanatory variables, or the lags of $y_{t}$; and β and $\delta_{j}\;(j=1,\cdots ,m+1)$ are the coefficients. $T_{i}\;(i=1,\cdots ,m)$ represents the index value of a node. The final structural breakpoints are determined by comparing the explanatory power of the whole regression equations.

## 4. Empirical Results

The log-returns of WTI crude oil futures, S&P 500 stock index futures and CSI 300 stock index futures were studied. According to the research process introduced in the previous section, the following results were obtained by estimating the VAR model, MIC, DCC–GARCH model and B-P (breakpoint) test.

### 4.1. WTI Crude Oil Futures and S&P 500 Futures

WTI crude oil futures and S&P 500 stock index futures were studied for full sample and phased samples. The following is for the overall sample, then the breakpoint test and finally the phased sample.

#### 4.1.1. Results for Overall Sample

Firstly, the Granger causality test was conducted on log-returns of WTI crude oil futures and log-returns of S&P 500 stock index futures. The following results were obtained:

Table 3 shows that log-returns of S&P 500 index futures are the Granger cause of log-returns of WTI crude oil futures at 1% significance level, while log-returns of WTI futures do not significantly Granger cause log-returns of S&P 500 futures. That means, on the whole, it is not the change of WTI crude oil futures that affects the change of S&P 500 index futures, but the change of S&P 500 index futures affects the change of WTI crude oil futures. Generally speaking, crude oil inevitably influences the economic development of a region. However, the results in Table 3 do not support this opinion. The main reason is that the American financial industry has too much influence and the crude oil consumed in America is mainly for people’s livelihood. Therefore, the economic development of the United States promotes economic development in the world, and its economic trend affects the global economic trend, thus further affecting the demand for crude oil and the price movement of crude oil.

Next, the VAR model was used to study the transmission relationship in mean between log-returns of WTI futures and S&P 500 futures. According to AIC criterion, the optimal lag order of VAR was determined to be order 1. The Table 4 shows the results of the VAR model.

The results of the VAR model further verified the results of Granger causality. That is, log-returns of S&P 500 futures have a positive impact on log-returns of WTI futures at the significance level of 10%. In other words, log-returns of S&P 500 futures directly affect log-returns of WTI futures. Further, allowing for the nonlinear relationship between log-returns of WTI futures and S&P 500 futures, the MIC was calculated according to Equation (6), viz $MIC\left(R_{t}^{wtif},\;R_{t}^{spf}\right)=0.1612871$, indicating that the two log-return series have a weakly positive relationship. This result is consistent with positive impact of log-returns of S&P 500 futures on log-returns of WTI futures.

**Figure 5:**
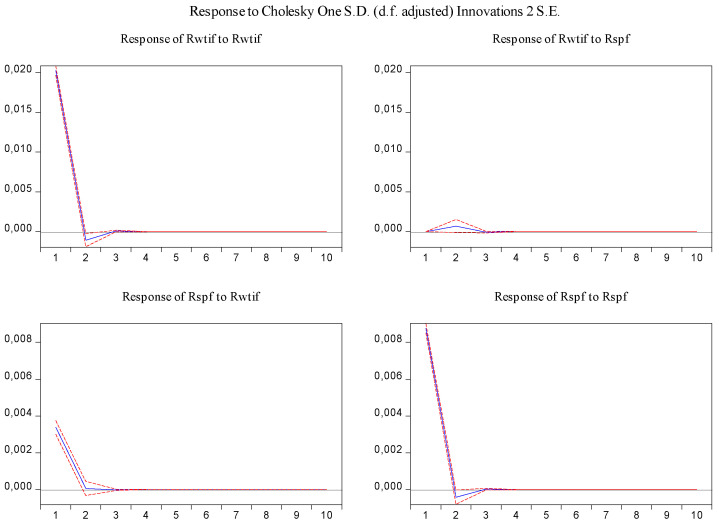
Impulse response of log-returns of WTI futures and S&P 500 futures in the overall sample.

In order to visually examine the response of variables to a shock or impulse from one of the other variables, the impulse response function was used for the above-estimated VAR model. From Figure 5, it can be observed that log-returns of WTI futures and S&P 500 futures react positively to their own past innovation in the first period and negatively in the second period, but after the third period, the response of log-returns of WTI futures and S&P 500 futures to their own past innovation dies out. In addition, shocks in log-returns of S&P 500 futures have no significant effect on log-returns of WTI futures, while shocks in log-returns of WTI futures have a statistically significant and positive effect on log-returns of S&P 500 futures in the first period, and then the effect disappears.

**Figure 6:**
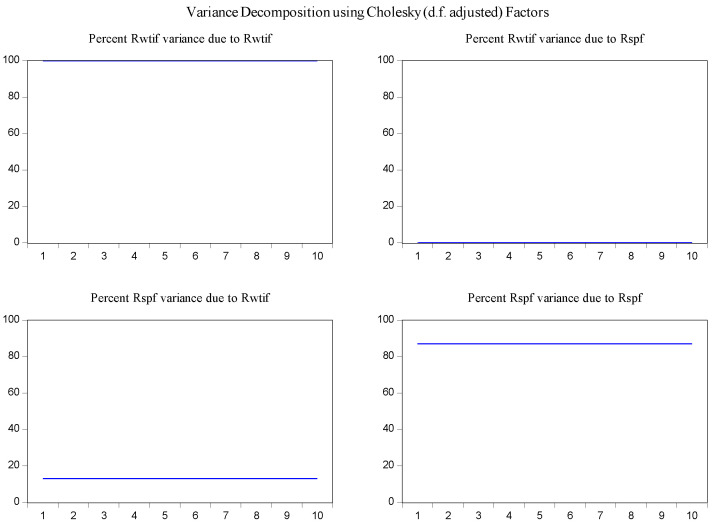
Variance decomposition of log-returns of WTI futures and S&P 500 futures in the overall sample.

In order to determine the source of the changes in the variables and to overcome the obstacles in the interpretation of the parameters in the VAR model [30], the variance decomposition method was used to examine the interaction of changes of log-returns of WTI futures on log-returns of S&P 500 futures. According to the results of variance decomposition for ten periods in Figure 6, the source of variance of log-returns of WTI futures almost entirely comes from their own shocks. It is also found that log-returns of WTI futures are the secondary source of the changes in the log-returns of S&P 500 futures with a share of about 13 percent.

In summary, although the order of lag 1 in log-returns of S&P 500 futures has a weak and positive impact on present log-returns of WTI futures according to Granger causality test and the estimated parameters in VAR model, an initial shock of log-returns of S&P 500 futures has a significant impact on log-returns of WTI futures according to impulse response function and variance decomposition. On the contrary, an initial shock of log-returns of WTI futures results in a significant and positive effect on log-returns of S&P 500 futures in the first period.

**Figure 7:**
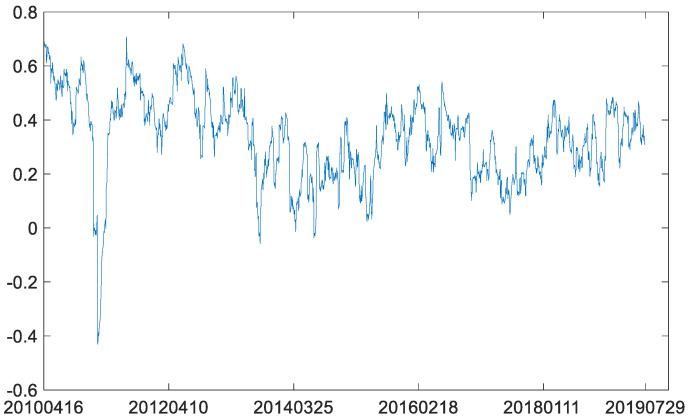
Coefficients of dynamic correlations between WTI futures and S&P 500 futures.

The residuals from the VAR model are then put into the DCC–GARCH model. As can be seen from Table 5, coefficients of DCC–GARCH reject the null hypothesis at the significance level of 1%, except that a constant term is significant at 5%. Moreover, the sum of DCC coefficients α and β is less than 1, indicating that the DCC model is stable. In addition, all coefficients in the GARCH model are significant; that is, residuals of the two futures markets have significant heteroscedasticity. According to the results of the DCC–GARCH model, the coefficients of dynamic correlations between WTI futures and S&P 500 futures can be obtained (Figure 7).

Figure 7 shows that coefficients of dynamic correlations between WTI futures and S&P 500 futures, as a whole, maintain low correlations after gradual decline. Crude oil prices have continued to rise as international supplies declined in the wake of the conflict in Syria. Because the virtual economy in the United States is decoupled from the real economy as a whole, there is no high positive correlation between the continuous rise of crude oil price and the American stock index, and even a negative correlation on 23 February 2011.

#### 4.1.2. Results for Phased Samples

Considering the structural changes in financial markets, the transmission relationship between WTI futures and S&P 500 futures in each phased sample, divided according to the B-P (breakpoint) test, are further analyzed. The B-P (breakpoint) test was used to find the location of the structural breakpoint. According to the BIC criterion (BIC has its minimum with 1 breakpoint and equals 16604), it can be determined that WTI futures have a structural breakpoint relative to S&P 500 futures, located on 25 November 2014. This structural breakpoint is broadly consistent with the OPEC’s announcement on 27 November 2014 that the daily production of crude oil would remain unchanged, meaning that the market had reacted well before the information was released. At the time, Petroleum Exporting Countries were widely expected to slash production to stabilize crude oil prices, which were volatile because of oversupply. However, information of maintaining production led to another sharp drop in crude oil prices. Next, the research process used in the overall sample is also applied to the two phased samples.

In line with Lin et al. [31], the Zivot and Andrews unit root test with a single structural breakpoint was used to examine stationarity of log-returns of WTI futures and S&P 500 futures. The Zivot and Andrews unit root test [32] represents an interesting alternative to the ADF test when there are breakpoints in the series. Statistics of the Zivot and Andrews unit root test for log-returns of WTI futures and S&P 500 futures are −52.0063 and −51.1431, respectively, rejecting the null hypothesis of a unit root at 1% significance level, thus indicating that the two log-return series are stationary. The phased Granger causality tests (Table 6) show that there is no significant Granger causality for either of the markets, except for a significant Granger causality of log-returns of WTI futures on S&P 500 futures in the first stage at 10% significance level. This reflects that the relationship in the mean between the US stock futures market and the crude oil futures market has weakened.

According to the AIC criterion, the lag order of VAR is 1. The VAR results are listed in Table 7. There is only a weak correlation between log-returns of WTI crude oil futures and log-returns of S&P 500 stock index futures in the first phased sample, which is consistent with results of the Granger causality test. Moreover, MICs in the two phased samples were calculated. It is obvious that the relationship between the two log-return series in the first phased sample is larger than that in the second phased sample. The results of MICs are similar to these in the VAR model and the Granger causality test, showing the decreasing correlation between WTI crude oil futures and S&P 500 stock index futures.

**Figure 8:**
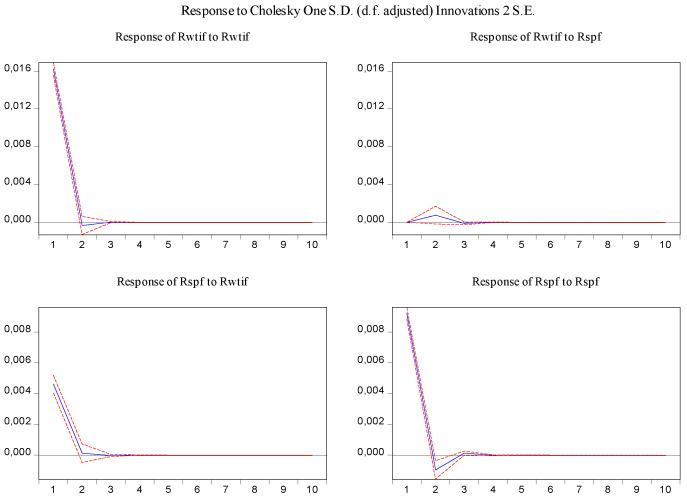
Impulse response of log-returns of WTI futures and S&P 500 futures in the first phased sample.

**Figure 9:**
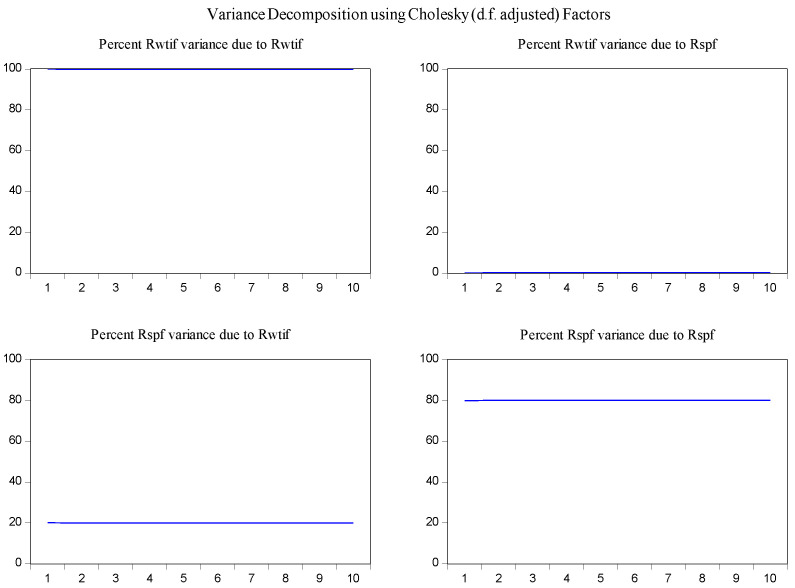
Variance decomposition log-returns of WTI futures and S&P 500 futures in the first phased sample.

**Figure 10:**
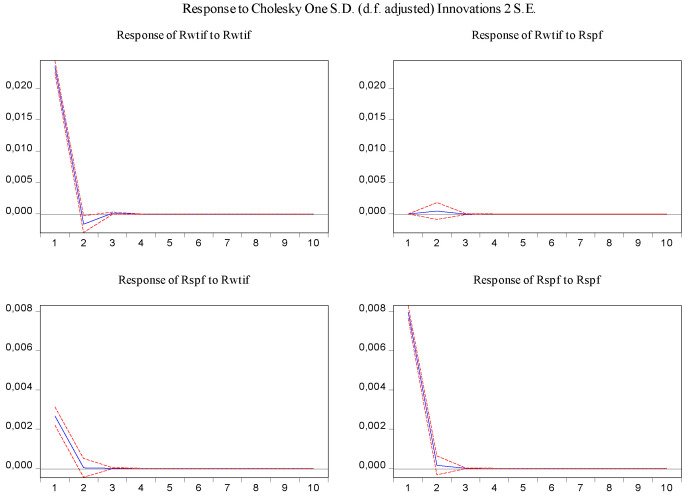
Impulse response of log-returns of WTI futures and S&P 500 futures in the second phased sample.

**Figure 11:**
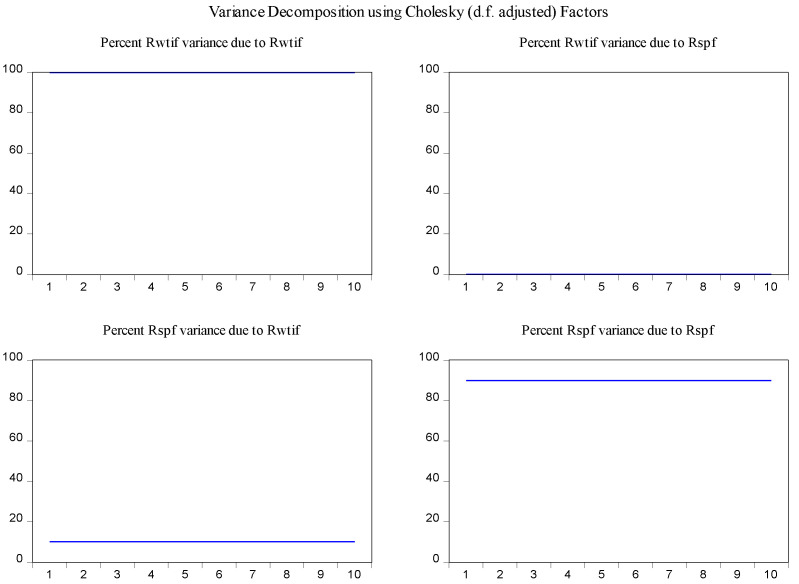
Variance decomposition of log-returns of WTI futures and S&P 500 futures in the second phased sample.

In the same fashion, the impulse response function and variance decomposition are conducted for the two phased samples of log-returns of WTI futures and S&P 500 futures. The results in Figure 8, Figure 9, Figure 10 and Figure 11 are similar to those in Figure 5 and Figure 6 and show the positive impact of log-returns of WTI crude oil futures on log-returns of S&P 500 stock index futures in the two phased samples and a weaker impact of the former on the latter in the second phased sample than in the first phased sample, which is consistent with results of the Granger causality test, the VAR model and the MICs.

**Figure 12:**
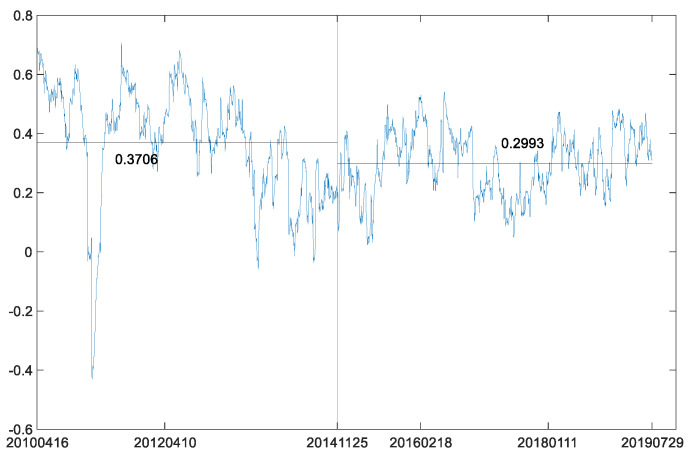
Coefficients of dynamic correlations between WTI futures and S&P 500 futures.

Next, the B-P (breakpoint) test was also used to analyze the structural break in the dynamic conditional correlation between the two futures markets. According to BIC criterion (BIC has its minimum with 0 breakpoint and equals −9957.954), there is no breakpoint in the dynamic conditional correlation between WTI crude oil futures market and S&P 500 stock index futures market. However, when we check the same breakpoint date of the price series in the dynamic conditional correlations, it is obvious that the average value of the conditional correlations before the breakpoint is larger than that after the breakpoint (Figure 12). This result is similar to the MICs in Table 7. The MIC in the second phased sample is less than that in the first phased sample. This further reflects the characteristics of decoupling of American stock index futures from crude oil futures.

### 4.2. WTI Crude Oil Futures and CSI 300 Futures

The research process in this section is the same as in Section 4.1; thus, the introduction of the models used in this section is omitted, and the relevant results are illustrated and analyzed.

According to the B-P (breakpoint) test (BIC has its minimum with 1 breakpoint and equals 16,265), it can be obtained that there is one structural breakpoint in WTI futures relative to CSI 300 futures, viz on 26 November 2014. Further, statistics of the Zivot and Andrews unit root test for the two log-returns are −50.6314 and −44.7218, respectively, also rejecting the null hypothesis of a unit root at 1% significance level, thus indicating that the two log-return series are stationary. Table 8 shows that, whether in overall sample or in each phased sample, log-returns of CSI 300 futures are affected by log-returns of WTI futures. These results are different from S&P 500 futures and WTI futures. There is a statistically significant Granger causality from WTI futures to CSI 300 futures. This is not hard to understand. As the world’s manufacturing factory, production activities of manufacturing enterprises account for the vast majority of China’s economic growth, so crude oil has a crucial impact on China’s economic growth. However, the Chinese economy is also in structural transformation, and the industrial structure is also gradually transforming into high-tech production and design activities. Crude oil is not the main determinant in these high-tech production activities. Therefore, the dependence of the Chinese economy on crude oil will be reduced. In addition, the use of new energy technologies has a certain impact on the traditional crude oil, which will further reduce the dependence of the Chinese economy on crude oil.

The results in Table 9 are consistent with the Granger causality test results. Log-returns of CSI 300 futures in the overall sample are significantly affected by log-returns of WTI futures, while the log-returns of CSI 300 futures do not affect log-returns of WTI futures. In the first phased sample, the log-returns of CSI 300 futures are significantly affected only by the log-returns of WTI futures, and the log-returns of WTI futures positively affect the log-returns of CSI 300 futures. In the second phased sample, this phenomenon still exists, but it is less significant than in the first phased sample. In addition, the MICs coincide with the results of VAR model and Granger causality test. There is a weak relationship between the log-returns of WTI futures and log-returns of CSI 300 futures in the overall sample, while the relationship between the two log-return series in the first phased sample is a little larger than that in the second phased sample, showing a gradually decreasing correlation between WTI crude oil futures market and CSI 300 stock index futures market.

**Figure 13:**
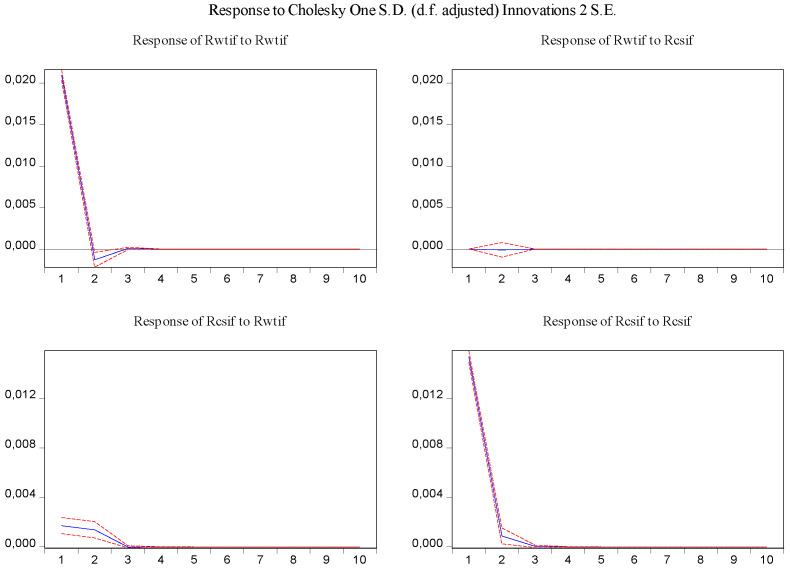
Impulse response of log-returns of WTI futures and CSI 300 futures in the overall sample.

**Figure 14:**
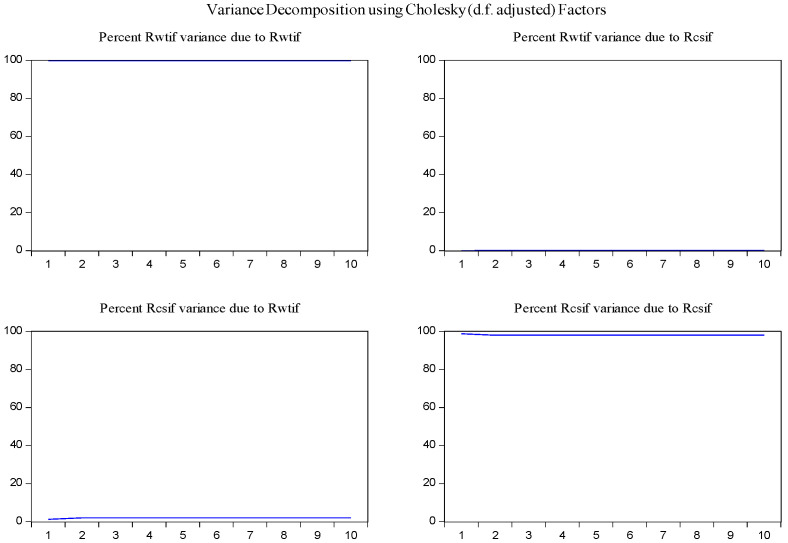
Variance decomposition of log-returns of WTI futures and CSI 300 futures in the overall sample.

**Figure 15:**
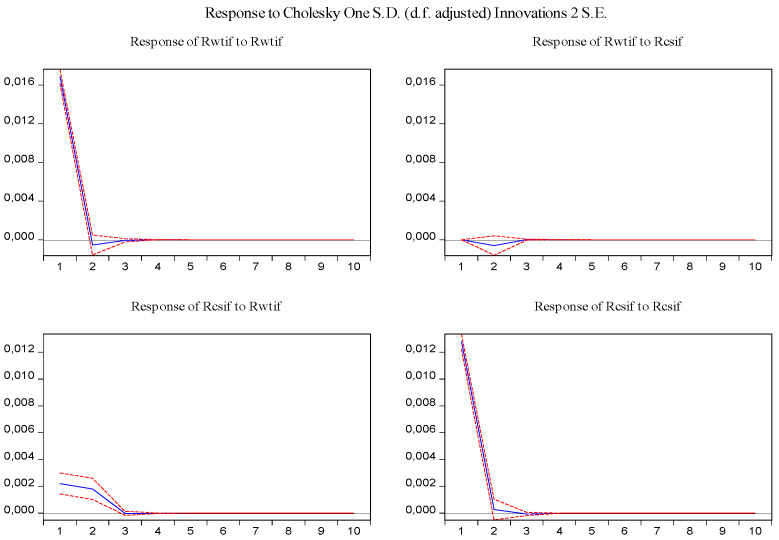
Impulse response of log-returns of WTI futures and CSI 300 futures in the first phased sample.

**Figure 16:**
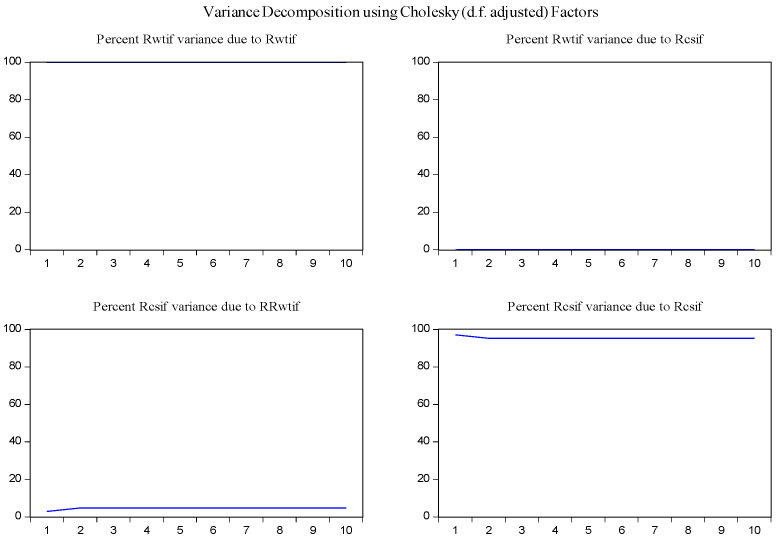
Variance decomposition of log-returns of WTI futures and CSI 300 futures in the first phased sample.

**Figure 17:**
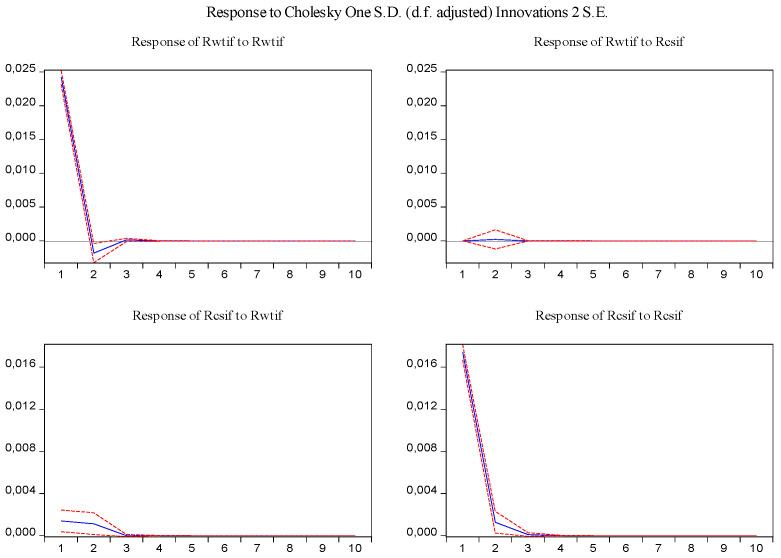
Impulse response of log-returns of WTI futures and CSI 300 futures in the second phased sample.

**Figure 18:**
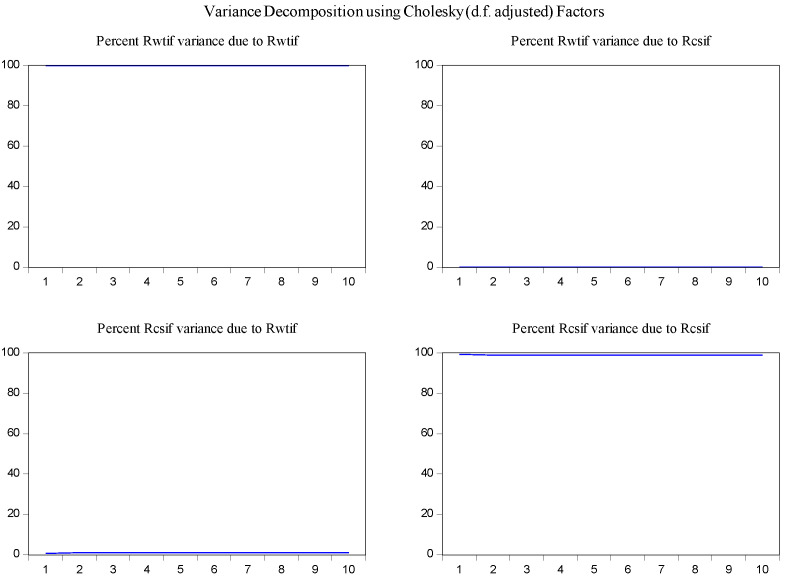
Variance decomposition of log-returns of WTI futures and CSI 300 futures in the second phased sample.

Furthermore, the impulse response function and variance decomposition are conducted for the overall and the two phased samples of log-returns of WTI futures and CSI 300 futures. From Figure 13, Figure 14, Figure 15, Figure 16, Figure 17 and Figure 18, it can be seen that the response of log-returns of WTI futures and CSI 300 futures on their own shock is statistically significant and positive in the beginning period, negative in the second period, and then disappears from the third period. An initial shock of log-returns of CSI 300 futures has no significant effect on log-returns of WTI futures, while an initial shock of the latter has significant and positive effect on the former in the first two periods, and then disappear from the third period. In addition, the response of log-returns of CSI 300 futures to the shock of log-returns of WTI futures is stronger in the first phased sample than in the second phased sample. Accordingly, the source of the changes in log-returns of WTI futures almost entirely comes from their own shocks, while log-returns of WTI futures are the secondary source of the changes in the log-returns of S&P 500 futures with a share of about 2 percent in the overall sample, 5 percent in the first phased sample and 1 percent in the second phased sample. These results show a gradually decreasing impact of log-returns of WTI futures on log-returns of S&P 500 futures and are consistent with the results in Table 9.

The results for DCC–GARCH model based on log-returns of WTI futures and CSI 300 futures in Table 10 are consistent with those based on log-returns of WTI futures and S&P 500 futures. All coefficients in the GARCH model are significant. However, it is worth noting that coefficients of DCC α in the overall sample are not significant. That is to say, the dynamic variances between log-returns of CSI 300 futures and WTI futures are mainly affected by the previous dynamic variances, while the short-term random fluctuations have little impact on them. This is different from the results between WTI futures and S&P 500 futures, because the economic environment in China is different from that in America. On the one hand, China has a strong ability to control the domestic crude oil price; on the other hand, the Chinese financial market is quite different from the American financial market. The Chinese financial market is weak and less efficient, while the American financial market is semi-strong and efficient. A variety of factors jointly lead to an insignificant impact of short-term fluctuations of crude oil prices on the Chinese financial market. However in the long run, fluctuations of crude oil prices will gradually affect the Chinese financial market.

**Figure 19:**
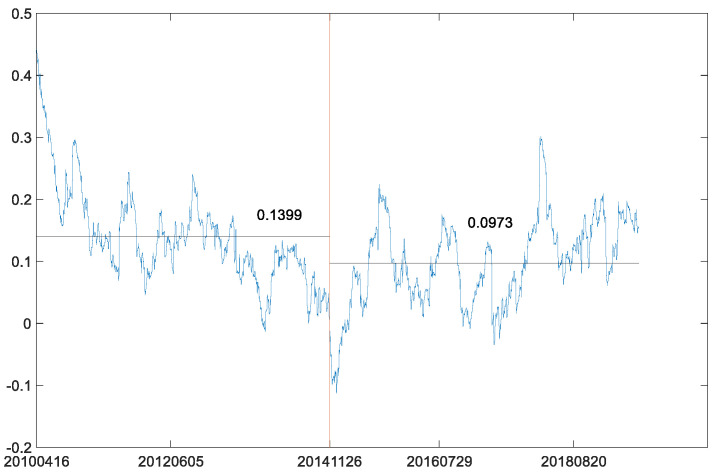
Coefficients of dynamic correlations between WTI futures and CSI 300 futures.

Then, the B-P (breakpoint) test was also conducted on the dynamic conditional correlation between log-returns of WTI futures and CSI 300 futures. According to the BIC criterion (BIC has its minimum with no breakpoint and equals −1.377 × 10^4^), there is no breakpoint in the dynamic conditional correlation between the two markets. Further, we also checked the same breakpoint date of the price series in the dynamic conditional correlations between the two log-return series. Our comparison of average values of the conditional correlations before the breakpoint and after the breakpoint (Figure 19) shows a significant decline from 0.1399 to 0.0973. These results coincide with the MICs in Table 9. Moreover, the dynamic correlation coefficients after the breakpoint are smoother than those in the overall sample. Therefore, it can be inferred that the Chinese economy will enter a period of prosperity when prices of crude oil fall. At the same time, the decline of correlation coefficients between crude oil futures market and Chinese futures market also shows that the influence of crude oil on Chinese economy is weakening. Therefore, the development of Chinese economy needs to fully consider the resource dividend brought by the decline of crude oil prices and is keeping a close eye on the issue of energy security. Although the correlation coefficients between Chinese futures market and crude oil futures market are decreasing, crude oil, as one of the most important energy sources, still plays a huge role in national security.

## 5. Conclusions

Combined with the B-P (breakpoint) test, VAR model and DCC–GARCH model, this paper studied the transmission relationship between the crude oil futures market and stock index futures market. The B-P (breakpoint) test can effectively describe the structural change between financial markets. Based on identification of structural breakpoints, the VAR model and the DCC–GARCH model were applied to describe the transmission relationship in the mean and the characteristics of coefficients of dynamic correlations between crude oil futures and stock index futures in each phased sample. In addition, the MIC was used to detect the nonlinear relationship between the two futures. This may provide more information than the indiscriminate application of the VAR–DCC–GARCH model on the overall sample. The findings are as follows.

Granger causality and the VAR model results show that, although S&P 500 futures are the Granger cause of WTI futures in the overall sample, the significance of coefficients in the VAR model is only at the 10% level. In the phased samples, there is no Granger causality between these two futures markets, and the coefficients of VAR model are no longer significant. In contrast, the Granger causality test and VAR model of CSI 300 futures and WTI futures pass the statistical tests at a high significance level both in the overall sample and phased samples. On the other hand, the dynamic correlation coefficients between WTI futures and S&P 500 futures/CSI 300 futures, on the whole, remain around 0.3 and 0.2, respectively. However, after the sharp drop in crude oil prices in 2014, the correlation coefficients between WTI futures and S&P 500 futures/CSI 300 futures experienced a significant decrease. This means that the breakpoint of the relationship in mean between WTI futures and S&P 500 futures/CSI 300 futures based on the B-P (breakpoint) test has a significant impact on the correlation of crude oil market and stock index futures markets.

The trend shows that the relationship of both the S&P 500 index futures and CSI 300 index futures with WTI crude oil futures is weakening. According to the Granger causality test in phased samples, the significance of the Granger causality of WTI futures relative to both American stock index futures and Chinese stock index futures in the first phased sample is higher than that in the second phased sample, viz one star (“*”). Moreover the results of the VAR model are similar. The MICs are in accord with the results of VAR model and Granger causality test. The MICs between WTI crude oil futures and S&P 500 index futures or CSI 300 index futures in the first phased sample are larger than those in the second phased sample. In other words, since the decline of crude oil prices in 2014, the transmission relationship in mean between WTI crude oil futures and both Chinese stock index futures and American stock index futures is weakening. Meanwhile, from the perspective of dynamic correlation coefficients, the overall dynamic correlation is also declining, which further confirms that the correlation between crude oil futures and stock index futures is gradually decreasing. This is mainly due to the fact that both China and the United States are in the process of industrial upgradation and transformation. This results in reduced importance crude oil in these economies and the resources they need for production. In addition, the emergence of all kinds of new energy alternatives has also exacerbated the trend.

Comparatively speaking, short-term fluctuations of WTI crude oil futures have less influence on Chinese stock index futures than American stock index futures. It can be seen from the DCC–GARCH model that the DCC α of CSI 300 futures and WTI futures is not significant. That is, short-term fluctuations of Chinese stock index futures and WTI crude oil futures do not impact each other. However, the coefficients in the DCC–GARCH model of the American stock index futures and WTI crude oil futures are significant. On the one hand, it is due to China’s strong ability to control the domestic crude oil price; on the other hand, it is due to the weak effective characteristics of the Chinese financial market. A variety of factors jointly lead to the short-term fluctuations of crude oil prices not having a significant impact on the Chinese financial market. However, in the long run, fluctuations of crude oil prices will gradually affect the Chinese financial market also.

## Figures and Tables

**Table 1:** Descriptive statistics for log-returns of WTI crude oil futures and S&P 500 futures.

Variable	$R^{wtif}$	$R^{spf}$
Mean	−0.0001583	0.0003871
SD	0.0203	0.0094
Kurtosis	5.6972	8.9260
Skewness	0.0365	−0.5726
Max	0.1162	0.0530
Min	−0.0907	−0.0750
JB stats.	730.17 ***	3653.5 ***
ADF	−51.7483 ***	−50.9143 ***
PP	−51.677 ***	−51.288 ***

*** indicates significance at 1% level.

**Table 2:** Descriptive statistics of log-returns of WTI crude oil futures and CSI 300 futures.

Variable	$R^{wtif}$	$R^{csif}$
Mean	−0.0001914	0.0000466
SD	0.0210	0.0156
Kurtosis	5.4464	12.6887
Skewness	0.0021	−0.6783
Max	0.1064	0.0953
Min	−0.0907	−0.1507
JB stats.	559.84 ***	8953.1 ***
ADF	−50.3592 ***	−44.5328 ***
PP	−50.301 ***	−44.488 ***

Note: *** indicates significance at 1% level.

**Table 3:** Results of Granger causality test on log-returns of WTI futures and S&P 500 futures.

Granger Causality Tests the Null Hypothesis	F Statistics
$R^{spf}$ is not the Granger cause of $R^{wtif}$	3.5684 ***
$R^{wtif}$ is not the Granger cause of $R^{spf}$	1.1039

Note: *** indicates significance at 1% level.

**Table 4:** Overall results of VAR model on log-returns of WTI futures and S&P 500 futures.

	C	$R_{t-1}^{wtif}$	$R_{t-1}^{spf}$
$R_{t}^{wtif}$	−0.0002	−0.0667 ***	0.0794 *
$R_{t}^{spf}$	0.00004 **	0.0110	−0.0477 **

Note: ***, **, and * indicate significance at 1%, 5% and 10% levels.

**Table 5:** Overall results of DCC–GARCH model based on the residuals of WTI futures and S&P 500 futures.

		Coefficients
$\varepsilon^{wtif}$	c	0.0000036 **
	$\varepsilon_{t-1}^{wtif}$	0.0616 ***
	$H_{t-1}^{wtif}$	0.9305 ***
$\varepsilon^{spf}$	c	0.0000037 ***
	$\varepsilon_{t-1}^{spf}$	0.1897 ***
	$H_{t-1}^{spf}$	0.7777 ***
	DCC α	0.0342 ***
	DCC β	0.946 ***

Note: *** and ** indicate significance at 1% and 5% levels.

**Table 6:** Phased results of Granger causality test on log-returns of WTI futures and S&P 500 futures.

F Statistics	First Phased Sample	Second Phased Sample
$R^{spf}$ is not the Granger cause of $R^{wtif}$	2.5922	0.5517
$R^{wtif}$ is not the Granger cause of $R^{spf}$	3.2558 *	0.0131

Note: * indicates significance at 10% level.

**Table 7:** Phased results of VAR model and MICs of WTI futures and S&P 500 futures.

		C	$R_{t-1}^{wtif}$	$R_{t-1}^{spf}$	MIC
First phased sample	$R_{t}^{wtif}$	−0.00013	−0.04354	0.08213	0.2022519
	$R_{t}^{spf}$	0.00052 *	0.03722 *	−0.10434 **	
Second phased sample	$R_{t}^{wtif}$	−0.00025	−0.07546 **	0.05972	0.1738922
	$R_{t}^{spf}$	0.00030	−0.00122	0.01883	

Notes: ** and * indicate significance at 5% and 10% levels.

**Table 8:** Results of Granger causality test on log-returns of WTI futures and CSI 300 futures.

F Statistics	Overall Sample	First Phased Sample	Second Phased Sample
$R^{csif}$→$R^{wtif}$	1.5475	1.4251	0.1041
$R^{wtif}$→$R^{csif}$	2.7761 **	19.231 ***	4.4003 **

Note: *** and ** indicate significance at 1% and 5% levels. $R^{csif}$→$R^{wtif}$ indicates $R^{csif}$ is not the Granger cause of $R^{wtif}$; $R^{wtif}$→$R^{csif}$ indicates $R^{wtif}$ is not the Granger cause of $R^{csif}$.

**Table 9:** Results of VAR model and MICs of WTI futures and CSI 300 futures.

		C	$R_{t-1}^{wtif}$	$R_{t-1}^{csif}$	MIC
Overall sample	$R_{t}^{wtif}$	−0.00019	−0.0609 ***	−0.006818	0.1105716
	$R_{t}^{csif}$	0.000086	0.0604 ***	0.0572 ***	
First phased sample	$R_{t}^{wtif}$	−0.00017	−0.02590	−0.04671	0.1371738
	$R_{t}^{csif}$	−0.00012	0.1043 ***	0.02130	
Second phased sample	$R_{t}^{wtif}$	−0.00023	−0.0752 ***	0.01223	0.1319133
	$R_{t}^{csif}$	0.00027	0.0426 **	0.07307 **	

Note: *** and ** indicate significance at 1% and 5% levels.

**Table 10:** Overall results of DCC–GARCH model based on the residuals of WTI futures and CSI 300 futures.

		Coefficients
$\varepsilon^{wtif}$	c	0.0000032 ***
	$\varepsilon_{t-1}^{wtif}$	0.0625 ***
	$H_{t-1}^{wtif}$	0.9321 ***
$\varepsilon^{csif}$	c	0.0000017 **
	$\varepsilon_{t-1}^{csif}$	0.0514 ***
	$H_{t-1}^{csif}$	0.9446 ***
	DCC α	0.0111
	DCC β	0.9731 ***

Note: *** and ** indicate significance at 1% and 5% levels.

## Data Availability

The daily close price data of the CSI 300 index futures was obtained from China Stock Market & Accounting Research Database (http://www.gtarsc.com/, accessed on 6 September 2021), while the daily close price data of the WTI crude oil futures and the S&P 500 index futures was obtained from Investing website (https://cn.investing.com/, accessed on 6 September 2021).
